# Comparative genome sequence and phylogenetic analysis of chloroplast for evolutionary relationship among *Pinus* species

**DOI:** 10.1016/j.sjbs.2021.10.070

**Published:** 2021-11-12

**Authors:** Umar Zeb, Xiukang Wang, AzizUllah AzizUllah, Sajid Fiaz, Hanif Khan, Shariat Ullah, Habib Ali, Khurram Shahzad

**Affiliations:** a Department of Biology, The University of Haripur, 22620, Pakistan; b College of Life Sciences, Yan’an University, Yan’an 716000, Shaanxi, China; c Department of Plant Breeding anf Genetics, The University of Haripur, 22620 Haripur, Pakistan; d Key Laboratory of Resource Biology and Biotechnology in Western China, Ministry of Education, College of Life Sciences, Northwest University, Xi’an 710069, China; e Department of Botany University of Malakand, Pakistan; f Department of Agricultural Engineering, Khawaja Fareed University of Engineering and Information Technology, Rahim Yar Khan, Punjab, Pakistan

**Keywords:** *Pinus*, Plastid genome, Sequence differentiation, Divergence time, Divergence hotspots

## Abstract

Genus *Pinus* is a widely dispersed genus of conifer plants in the Northern Hemisphere. However, the inadequate accessibility of genomic knowledge limits our understanding of molecular phylogeny and evolution of *Pinus* species. In this study, the evolutionary features of complete plastid genome and the phylogeny of the *Pinus* genus were studied. A total of thirteen divergent hotspot regions (*trnk-UUU, matK, trnQ-UUG, atpF, atpH, rpoC1, rpoC2, rpoB, ycf2, ycf1, trnD-GUC, trnY-GUA,* and *trnH-GUG*) were identified that would be utilized as possible genetic markers for determination of phylogeny and population genetics analysis of *Pinus* species. Furthermore, seven genes (*petD, psaI, psaM, matK, rps18, ycf1,* and *ycf2*) with positive selection site in *Pinus* species were identified. Based on the whole genome this phylogenetic study showed that twenty-four *Pinus* species form a significant genealogical clade. Divergence time showed that the *Pinus* species originated about 100 million years ago (MYA) (95% HPD, 101.76.35–109.79 MYA), in lateral stages of Cretaceous. Moreover, two of the subgenera are consequently originated in 85.05 MYA (95% HPD, 81.04–88.02 MYA). This study provides a phylogenetic relationship and a chronological framework for the future study of the molecular evolution of the *Pinus* species.

## 1 Introduction

*Pinus L*. (Pinaceae) is major coniferous genus consisted of more than (110–120) species. Because of its divergence and significant ecological value, the genus *Pinus* is the best option for the study of species divergence and evolution of conifers (Farjon, 1990, Neale and Kremer, 2011). These species are distributed throughout the world but it is the main coniferous genus of the northern hemisphere, which harbored over, Asia, Europe, North Africa, and Central America (Price et al., 1998). The genus Pine is originated in the mid-Cretaceous period, which is further diverged into two lineages, i.e. the subgenus *Strobus* (Haploxylon) and subgenus *Pinus* (Diploxylon) (Willyard et al., 2007, Millar, 1998). These species are ecologically essential assisting forest ecosystems and are economically very important for being used as fuel and timber (Ennos, 2001, Vekemans and Hardy, 2004). The anatomical, morphological, and evolutionary level data determine that the two subgenera are significantly separated (Wang et al., 1999, Gernandt et al., 2001). Generally, a valuable fossil record, of pine species divergence and later time calibrations have been used for the fewer fossils records (Gernandt et al., 2005, He et al., 2016, Moore et al., 2007). Further, the fossils records are contentious concerning their phylogenetic position and age limit.

There are several other techniques i.e., fossil records, haplotype investigation, time-calibrated phylogeny and DNA duplication etc. taken place to study the evolutionary relationship among Pine species. However, Next-generation sequencing technologies, utilizing the paternally inherited plastid DNA is a reliable tool to investigate the evolutionary and phylogenetic relationships in plants (Bentley et al., 2008, Langmead et al., 2009, Wilson et al., 2017)*.* Plastid genome has a particular genetic system, and perform a significant role in the photosynthesis (Ravi et al., 2008). Generally, chloroplast genome (cp genome) is circular DNA molecules, which classically have a quaternary molecular structure containing inverted repeats (IRa/IRb) regions, detached through single large copy (LSC) and small single copy (SSC) regions (Palmer, 1991, Asaf et al., 2017). However, the plastome round structure composed of four intersections in inverted repeat regions and the single-copy regions which hampered our capability to maintain exact chloroplast genome assemblies (Chin et al., 2013, Bashir et al., 2012). Previous studies showed that chloroplast genomes of gymnosperm species were more preserved in their gene structure, order and contents (George et al., 2015). Typical structure of cp genome of a majority of the land plants is spherical with a length of (120–160 kb), consist of (110–130) genes (Ruhlman and Jansen, 2014, Civáň et al., 2014). The complete chloroplast DNA sequences of closely related species confides several evolutionary hotspots region for mutations in the whole chloroplast genomes of *Pinus* species. Phylo-genomics study provides an excessive ability to determine historically severe issues in phylogeny by decreasing sampling mistake (Lindgren and Anderson, 2018). Using different datasets of plastid genomes the land plants showed different reconstructing phylogenetic tree at different taxonomic level (Luo et al., 2016, Zhang et al., 2017).

Plastid genome is identified in the plant phylogeny, evolution, and divergence of a species. Some works supported that phylogenetic analyses not only determine the previously discussed phylogeny but also increase accurate phylogenetic trees (Irisarri et al., 2017, Sass et al., 2016, Bravo et al., 2019). Nowadays, such type of studies is essential to point out the difference between various tree-building methods used for phylogenetic evaluations based on systematic errors. However, the systematic mistake will be eliminated by improving the dataset, which leads to improving the size of data (Crawford et al., 2012). Comparative study of related species with distinct environmental necessities and evolutionary histories can reveal insight into the mechanisms of the structural genetic adaptation (Ahmad et al., 2021). Comparative studies of the whole plastome are conducting to study the adaptive evolution of the genus *Pinus* showing differences in demographic history populations genetics, environmental conditions, or phylogenetic relationships (Grivet et al., 2013). The forest trees, adaptive evolution is difficult, throughout their life sequence. Moreover, because of the large size of the plastid genome, the comparative genomic studies of the forest trees are difficult. Recently, in-plant genomics divergence for sorts of spots that are anticipated to evolved inversely (synonymous and nonsynonymous). Meanwhile, positive selection has an impact on the plant morphology and phenology; more genes elaborate in these adaptations are still mostly unidentified. However, concern to gymnosperm species knowledge is inadequate. Positive site or complementary selection have been recognized for some selected genes (Eveno et al., 2008). *Pinus* life cycle provides excellent chances for robust selection. The gene flow in most of the plant population is higher, which make the selection in a well-organized manner. This study was conducted with the following specific objectives: (a) investigation of variation in the gene order, gene content and repetitive sequence in whole plastid genomes of *Pinus* species (b) to recognize the hotspots region of chloroplast genomes and to find out the possibility under selection pressure (c) to recreate molecular divergence and phylogeny within the main ancestries of *Pinus* species.

## 2 Materials and methods

### 2.1 Materials

The whole plastid DNA dataset of twenty-four genus *Pinus* and the out groups were found from the NCBI (https://www.ncbi.nlm.nih.gov/). We also re-annotated the *Pinus* complete chloroplast genomes sequenced for the analysis.

### 2.2 Chloroplast genome Sequencing, Annotation, and divergence analysis

The chloroplast genomic data were used to generate a consensus sequence inside the Geneious R v 8.0.2 (Biomatters Ltd., Auckland, New Zealand). The preliminary plastome annotation was turned using program DOGMA (https://dogma.ccbb.utexas.edu/). The stop and start codons were adjusted manually in the Geneious R v 8.0.2. The Organellar Genome DRAW v1.1 (OGDRAW) utilized for construction of circular plastid cp genome map (Wyman et al., 2004, Lohse et al., 2007). For the divergence sequence in the *Pinus* plastome, the sequence reorganization analysis of the *Pinus* genome was used (Morse et al., 2009), and *Pinus* species were determined through mVISTA (Frazer et al., 2004), as used for the investigation of *P. bungeana* as a reference.

### 2.3 Repeat sequence and selective pressure analysis

Repeat sequence analysis is handy markers which possess dynamic roles in the phylogenetic analysis and evolutionary studies (Ni et al., 2017). We find the three repeats’ sequences i.e., dispersed, palindromic, and tandem, and the web-based software REPuter (https://bibiserv.cebitec.uni-bielefeld.de/reputer) was used to investigate the repeat sequences (Kurtz et al., 2001). The dispersed and palindromic repeated sequences are (a) sequence identity 90%; (b) Hamming distance = 1; and (c) minimum repeat size = 30 bp (Benson, 1999). Moreover, the tandem motifs examination (>10 bp in length) was identified using the Tandem Repeats Finder program (https://tandem.bu.edu/trf/trf.html). We examined the repeat sequence manually in the cp DNA of twenty-four *Pinus* species with the genomic sequence, simple sequence repeats (SSR) through the Perl script MISA program (http://pgrc.ipk-gatersleben.de/misa/). The three repeat units for mono-, di-, tri-, tetra-, penta-, and hexa nucleotide SSRs respectively (Thiel et al., 2003).

The Codeml program (http://nebc.nerc.ac.uk/nebc_website_frozen/nebc.nerc.ac.uk//index.html) was employed to understand the codon-substitution models, PAML package v 4.7.1 (http://abacus.gene.ucl.ac.uk/software/paml.html) for analysis of synonymous (dS) and non-synonymous (dN) nucleotide substitution rates, along with their ratios (ω = dN/dS) (Yang, 2007). The Geneious R v 8.0.2 was employed for identification and alignment of protein-coding gene (Stamatakis, 2014). Protein-coding exon and each value of dN; dS, and ω were calculated using the site-specific model apply in the codeml package (seqtype = 1, model = 0, Nsites = (0, 1, 2, 3, 7, 8) in PAML4.7 (Yang et al., 2005). Generally, this model permissible the ω proportion to be different among sites with a settled ω ratio have evolution in the site-specific gene phylogeny (Katoh and Standley, 2013). To determine the assistance of selected sites, we compared the modal site-specific M0 (one ratio) vs M3 (discrete), M1 (neutral) vs M2 (positive selection), M7 (beta) vs M8 (beta and ω), were related in site-specific models (Katoh and Standley, 2013). The Model M1 was used to determine two site classes with ω < 1 and ω = 1 and model M2 was used to examine the third side class ω > 1. The M7 and M8 model equally explained the ω circulate as a beta function. The model M7 beta null limitation ω to (0, 1), the substitute beta and ω model M8 used for other selected site classes. Only consistent sites of positive selection with important from posterior probability (p (ω > 1 ≥ 0.99) were identified; Modal M2 and M8 recognized Bayes Empirical Bayes approach (BEB) were further considered.

### 2.4 Phylogenetic analysis

The evolutionary relationship among the available complete chloroplast genome of twenty-four *Pinus* species were utilized to reconstruct the phylogenetic tree. We also include cp genome sequences from *Cupressus gigantean* (KT315754) and *Cupressus chengiana* (KY392754) as out-groups. Plastid plastome of *Pinus* species from the complete dataset were aligned with MAFFT v 7.0.0 (Yang and Nielsen, 20022002), after that nucleotide sequence alignment were performed with the Clustal W technique using the MEGA v 7.0.18 (Tamura et al., 2007), with manual inspection. However, maximum likelihood (ML) and maximum parsimony (MP) evaluated the inferred evolutionary trees, implemented the best-fit modal of the cp genome sequence evolution preferred by Model Test version 3.7 with the Akaike Information Criterion (AIC) (Posada et al., 20042004). The phylogenetic tree was assessed by (1000) bootstrap value. It was then used to approximate MP and ML tree branch support values. The best phylogenetic model was determined through PAUP* (Swofford, 2003). In addition, the Bayesian phylogenetic analysis was performed by MrBayes v3.1.2. Markov chain Monte Carlo (MCMC) investigation was commenced from an arbitrary tree and run for 3,000,000 generations with the experiment of topologies for every (1000) generation (Ronquist and Huelsenbeck, 2003). Subsequently, the initial 2500 trees (corresponds to 25% of our samples) were removed as burn-in (as suggested by the manual of MrBayes). Further, the trees were used to build 50% more-rule consensus tree and inferring Bayesian posterior probabilities of the nodal supports. The output was assessed using the FigTree v 1.3.1 (Rambaut, 2010).

### 2.5 Divergence time analysis

The BEAST v.2.4.5 software was used for the divergence time estimation which estimated the node ages and topology (Bouckaert et al., 2014). The average substitute rate of 5 × 10^7^ s/s/y to calibrate the molecular divergence. The nucleotide substitutions of the GTR model and applied the ‘Bayesian skyline’ tree process model used with a standard normal prior. However, we set an ‘exponential relaxed clock’ with the previous substitution rate. Generally, the divergence times were assessed by Markov Chain Monte Carlo (MCMC) examination run for (30,000,000) generations. We tested 3000 trees with the preliminary 25% treated as burn-in, the tree provides a central 95% range of 85 Mya, within the ranges described by the two other analysis (Gernandt et al., 2008, Pennington et al., 2004) from the independent fossil calibrations. To check the chain balancing the results of MCMC was analyzed by Tracer v 1.5 programs. After that, the Tree Annotator v 1.7.5 program was used to get a good quality tree merging. The Figtree v 1.3.1 was used to clearly show the tree result (Pennington et al., 2004).

## 3 Results

### 3.1 Characteristics of twenty-four complete plastid genomes of Pinus species

**Fig.1:**
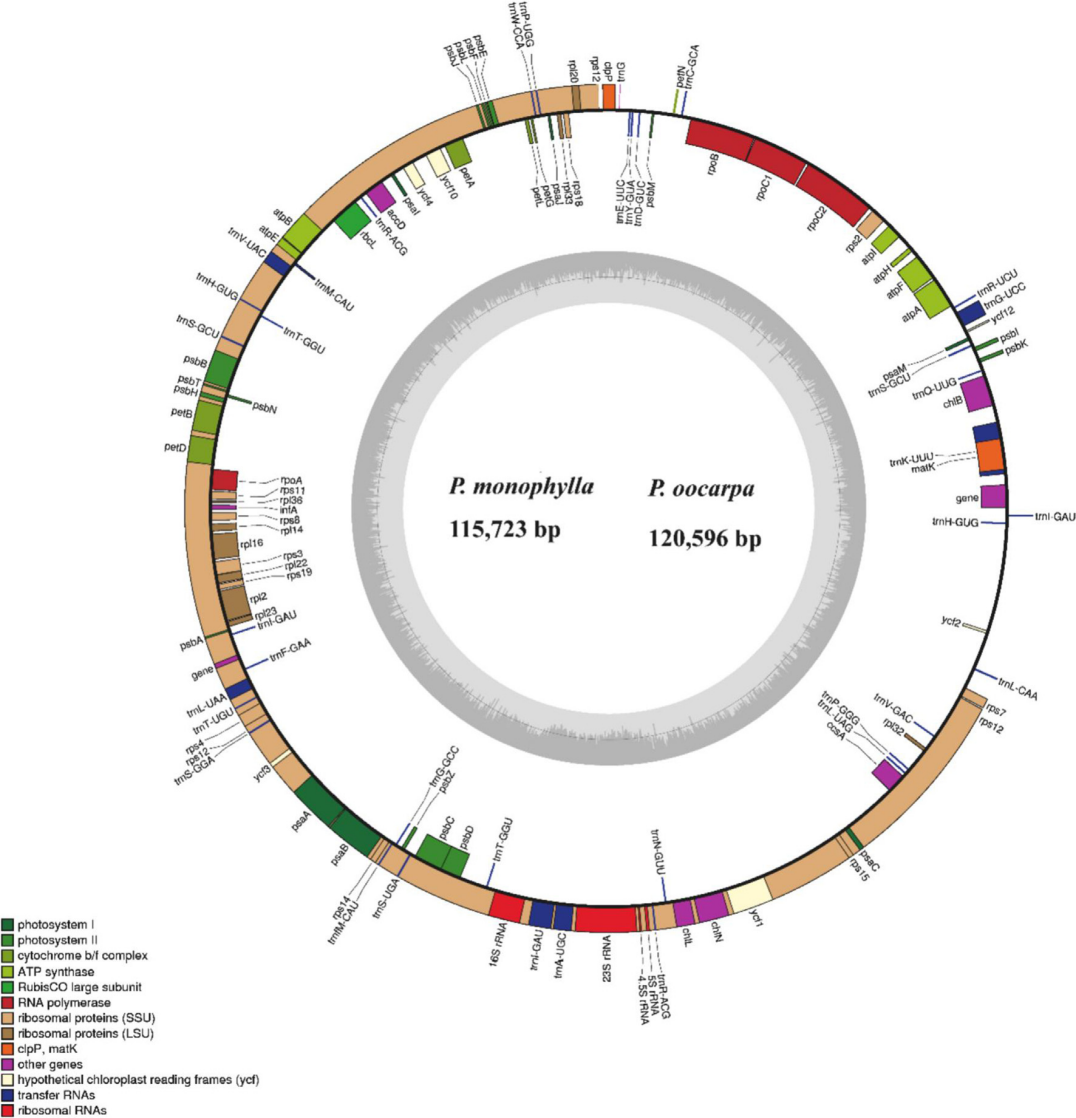
Sequence alignment of plastid genomes in 24 *Pinus* species. mVISTA-based identity plots show the identity between the chloroplast genomes of 24 *Pinus* species.

The comparison of full length and size of complete plastid DNA of twenty-four species of the genus *Pinus*, ranged from 115,723 bp (*P. monophylla*) to 120,596 bp (*P. oocarpa*) (Table 1, Fig. 1). These plastid DNA contains distinctive quadripartite circular structure, comparable to those in higher plants. In addition, the chloroplast genome of twenty-four *Pinus* species were divided into two different sections that coordinated to subgenus *Strobus* and subgenus *Pinus*. The subgenus *Strobus* size ranged from 116,119 bp (*P. krempfii*) to 117,805 bp (*P. fenzeliana*), and subgenus *Pinus* ranged in size from 115,909 bp (*P. oocarpa*) to 120,596 bp (*P. jaliscana*) (Table 1). The subgenus *Pinus* had an LSC region ranged from 64,415 bp (*P. sylvestris*) to 65,724 bp (*P. oocarpa*), and SSC region ranged from 50,661 (*P. sylvestris*) to 54,146 bp (*P. taeda*). The subgenus *Strobus,* the inverted repeats (IRs) region ranged from 326 bp (*P. sibirica*) to 516 bp (*P. gerardiana*), and subgenus *Pinus* from 389 bp (*P. greggii*) to 487 bp (*P. taiwanensis*) (Table 1). The complete chloroplast genome was composed of 114 functional genes, counting 74 protein-coding genes (CDS), four ribosomal RNA genes (rRNA), and 36 transfer RNA genes (tRNA). In the LSC region, 17 tRNA genes and 53 protein-coding genes were present, whereas the SSC region includes 17 tRNA genes and 18 protein-coding genes. Additionally, the *trnI*-GAU genes were repeated in the IRs region. Moreover, the total GC content was similar in the twenty-four genomes of *Pinus* species at about 38.6%. The overall GC content was irregularly circulated across the plastid DNA, which was highest in the SSC region (39.9%), followed by IRs (39.6%) and LSC (38.1%) regions (Table S1).

**Table 1:** The features of complete chloroplast genomes of twenty-four *Pinus* species.

**Section**	**Species**	**Size (bp)**	**LSC (bp)**	**SSC (bp)**	**IRs (bp)**	**Number of Protein Coding Genes**	**Number of rRNA Genes**	**Number of tRNA Genes**	**GC Contents (%)**	**Accession number**
**Subgenus *strobus* (single needle sections)**										
	*P. armandii*	116,998	64,337	51,711	389	75	4	36	37	NC_029847
	*P. bungeana*	116,751	64,311	51,490	475	75	4	36	38.8	NC_028421
	*P. fenzeliana*	117,805	64,490	52,565	375	75	4	35	36.8	KX255674
	*P. gerardiana*	116,668	64,296	51,339	516	75	4	36	38.7	EU998741
	*P. koraiensis*	116,781	64,337	51,494	475	76	4	36	38.8	AY228468
	*P. krempfii*	116,119	64,463	50,912	356	74	4	34	38.8	EU998742
	*P. lambertiana*	116,958	64,604	51,592	379	75	4	35	38.8	EU998743
	*P. monophylla*	115,723	64,299	50,664	373	73	4	36	38.7	EU998745
	*P. nelsonii*	116,210	64,604	50,845	367	74	4	35	38.7	EU998746
	*P. pumila*	117,398	64,606	51,842	384	75	4	36	38.0	JN854168
	*P. sibirica*	117,035	64,598	51,787	326	79	4	33	38.7	NC_028552
	*P. strobus*	116,975	64,286	51,827	474	75	4	36	38.8	NC_026302
	*P. longaeva*	117,726	65,107	51,665	482	74	4	36	38.6	–

**Subgenus *Pinus* (Double needle section)**										
	*P. greggii*	119,480	64,849	53,853	389	74	4	36	38.5	NC_035947
	*P. oocarpa*	120,596	65,724	54,089	394	73	4	36	38.5	KY963969
	*P. taeda*	120,534	65,610	54,146	389	75	4	36	38.5	NC_021440
	*P. contorta*	119,452	64,914	53,556	486	74	4	35	38.5	EU998740
	*P. massoniana*	119,025	65,139	53,108	389	75	4	36	38.6	NC_021439
	*P. sylvestris*	115,909	64,415	50,661	420	75	4	37	38.6	KR476379
	*P. mugo*	119,042	64,938	53,123	404	75	4	36	38.5	KX833097
	*P. thunbergii*	118,893	65,210	52,885	399	74	4	36	38.5	FJ899562
	*P. tabuliformis*	118,969	65,196	52,975	399	75	4	36	38.5	NC_028531
	*P. taiwanensis*	119,013	64,959	52,985	487	80	4	36	38.5	NC_027415
	*P. jaliscana*	119,697	64,805	54,092	403	75	4	37	38.5	NC_035948

Among 114 functional genes, 63 were linked to self-replication (36 in tRNA and 4 in rRNA), 9 were associated to large subunits of the ribosome, and 11 were associated to small subunits of the ribosome, and 4 genes were associated with DNA-dependent in RNA polymerase subunits. The *infA* gene was associated with the translational initiation factor. Subsequently, 40 genes were related with photosynthesis, six with ATP synthase, 6 genes with subunits of cytochrome, 11 genes with subunits of photosystem I and 8 genes with subunits of Photosystem II. Generally, about five extra genes were identified. However, the *matk* gene encoding Maturase, *accD* encoding subunit of acetyl-CoA, *ccsA* encoding C-type cytochrome synthesis gene, and *clpP* encoding Protease (Table 2). In the chloroplast genome, six genes (*trnS-GCU, trnI-GAU, trnS-UGA, trnH-GUG, trnT-GGU, trnR-ACG*) were repeated in all the *Pinus* plastomes.

**Table 2:** Gene contents in twenty-four *Pinus* species complete chloroplast genomes.

**Gene group**	**Gene name**				
Ribosomal RNA genes	*rrn16*	*rrn23*	*rrn4.5*	*rrn5*	
Transfer RNA genes	*trnI-CAU*	*trnI-GAU(rep)*	*trnL-UAA*	*trnL-CAA*	*trnL-UAG*
	*trnR-UCU*	*trnR-ACG(rep)*	*trnA-UGC*	*trnW-CCA*	*trnE–UUC*
	*trnV-UAC*	*trnV-GAC*	*trnF-GAA*	*trnT-UGU*	*trnT-GGU(rep)*
	***trnP-UGG***	*trnfM-CAU*	*trnP-GGG*	*trnG-GCC*	*trnS-GGA*
	*trnS-UGA(re)*	*trnS-GCU(rep)*	*trnD-GUC*	*trnC-GCA*	*trnN-GUU*
	*trnE-UUC*	*trnY-GUA*	*trnQ-UUG*	*trnK-UUU*	*trnH-GUG(rep)*
	*trnG-GCC*	*trnM-CAU*	*trnG-UCC*	*trnI-GAU*	
Small Subunit of ribosome	*rps2*	*rps3*	*rps4*	*rps7*	*rps8*
	*rps11*	*rps12*	*rps14*	*rps15*	*rps18*
	*rps19*				
Large Subunit of ribosome	*rp12*	*rp114*	*rp116*	*rp120*	*rp122*
	*rp123*	*rp132*	*rp133*	*rp136*	
DNA-dependent RNA polymerase	*rpoA*	*rpoB*	*rpoC1*	*rpoC2*	
Translational initiation factor	*infA*				
Subunits of photosystem I	*psaA*	*psaB*	*psaC*	*psaI*	*psaJ*
	*psaM*	*ycf1*	*ycf2*	*ycf3*	*ycf4*
	*ycf10*				
Subunits of photosystem II	*psbA*	*psbB*	*psbC*	*psbD*	*psbE*
	*psbF*	*psbH*	*psbI*	*psbJ*	*psbL*
	*psbM*	*psbN*	*psbT*		
				
Subunits of cytochrome	*petA*	*petB*	*petD*	*petG*	*petL*
	*petN*				
Subunits of ATP synthase	*atpA*	*atpB*	*atpE*	*atpF*	*atpH*
	*atpI*				
Large subunit of Rubisco	*rbcL*				
Maturase	*matk*				
Protease	*clpP*				
Subunit of acetyl-CoA	*accD*				
C-type cytochrome synthesis gene	*ccsA*				

### 3.2 Repetitive sequence analysis

**Fig. 2:**
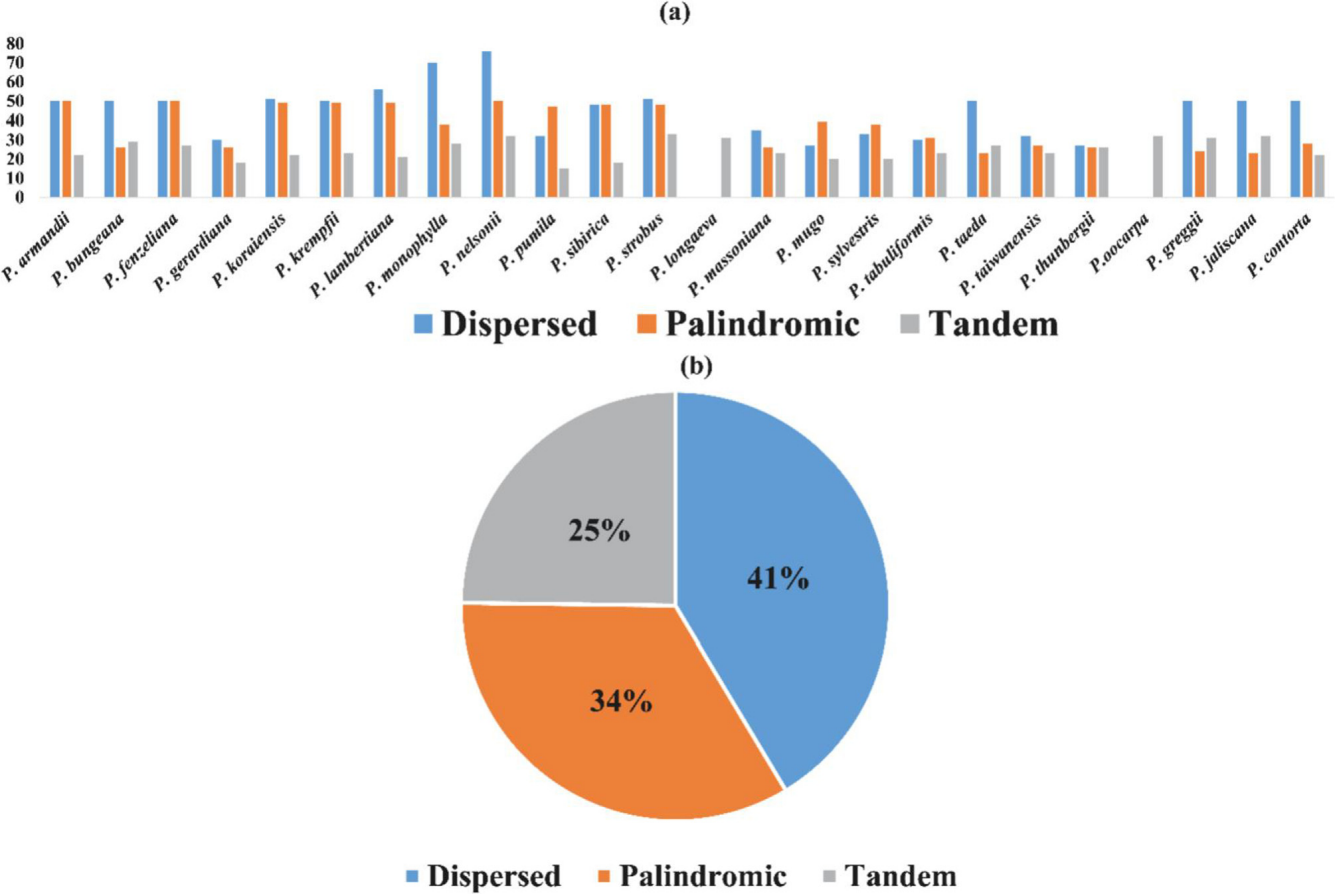
Repeat analyses. (a) Histogram showing the number of repeats in the twenty-four *Pinus* chloroplast genomes.

**Fig. 3:**
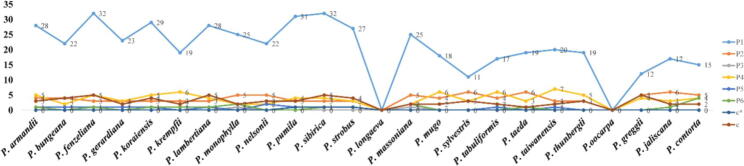
Simple sequence repeats (SSRs) in chloroplast genomes of the genus *Pinus.*

The investigation unearth three types of repeats (palindromic, dispersed and, tandem repeats) in complete chloroplast genomes of the twenty-four *Pinus* species*.* However, a sum of 2411 repeat units were identified in the whole plastome of genus *Pinus*, comprised of 998 (41%) dispersed repeats, 815 (34%) palindromic repeats, and 598 (25%) tandem repeats (Fig. 2). However, the dispersed repeats were more than palindromic repeats, and the tandem was minimum in *Pinus* species. Among various species, the number of repeats for *P. nelsonii* (76) and *P. pumila* (15) were the highest and lowest number respectively. We recognized a total of 769 SSR loci in the twenty-four *Pinus* plastids genomes (Fig. 3). Among these genes, the most common were mono-nucleotides repeats, about (4.91% of total SSRs), followed by di-nucleotides (0.89%) the tetra-nucleotide repeat number was more than tri-nucleotide repeats; the penta- and *hexa*-nucleotides were very less in all *Pinus* genome. Interestingly, most SSRs number originated in *P. sibirica* and *P. fenzeliana* (47, 47), and the *P. sylvestris* has the lowest number of repeats (23) (Fig. 3). We observed that almost all of the simple sequence repeats (SSR) were same in the recently sequenced *Pinus* species.

### 3.3 Divergence hotspot regions

To illuminate the level of genomic divergence, the sequence character among *Pinus* chloroplast DNA was determined using the mVISTA software as a reference with *P. bungeana* (Fig. S1). The analysis of this correlation showed that the divergence of IRs region is less than the SSC and LSC regions. Thus, the noncoding regions showed more variation than the coding regions, and profoundly variable regions among the *Pinus* plastome happen in the intergenic spacers. Interestingly, we identified that eleven genes positioned in LSC and SSC region within the coding and non-coding regions (*trnG-GCC,* trnL-UAG, *trnL-CAA*, *trnQ-UUG*, *rpoC1*, *rpoC2*, *psaC*, *ycf1*, *ycf2*, *chIL*, *chlN*), which showed a high level of variation as divergent Hotspot regions (Fig. S1).

### 3.4 Adaptive evolution analysis

The selective pressure analysis of chloroplast genomes of *Pinus* species for protein-coding genes was performed through the codon substitution models to scrutinize positive selection for potential sites. Seven genes with the positive selection site in twenty-four *Pinus* species (Table S2). Interestingly, all these were associated with the photosynthesis process, e.g., four genes (*psaI*, *psaM*, *ycf1*, and *ycf2*) encoded the subunits of photosystem I, one gene *rps18* was related to the small subunit of ribosome protein, one gene *petD* related to subunits of cytochrome *b*/f complex, and another *matK* was maturase. Also, *ycf1* and *ycf2* gene regions harbored above 100 sites under positive selection, followed by some *psaM* (16, 22), *rps18* (55) and the other genes (1, 1) had only one active site within modal M2 and M8 respectively (Table S2).

### 3.5 Phylogenetic relationship of genus Pinus

**Fig. 4:**
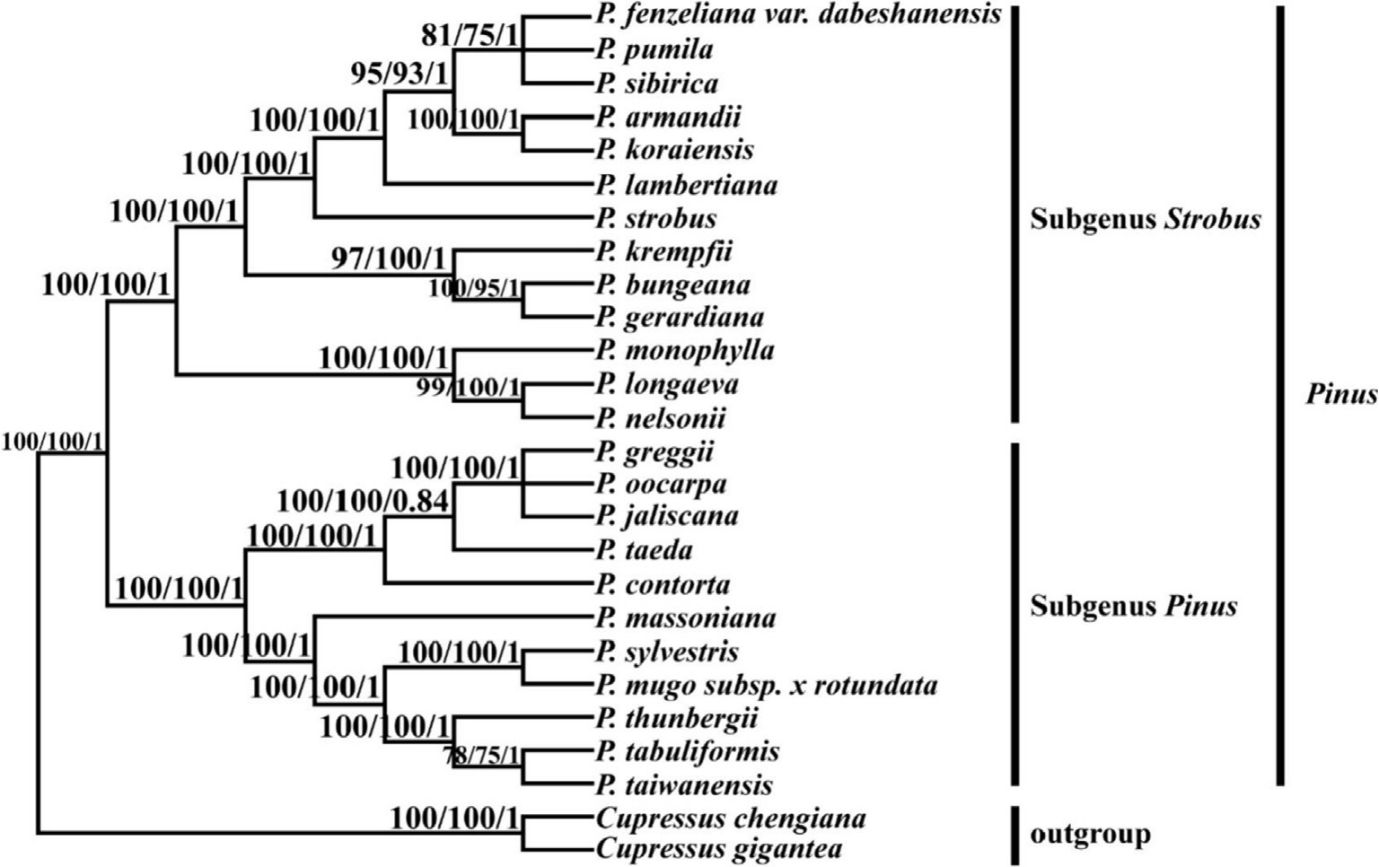
Phylogenetic tree obtained for twenty-four *Pinus* species based on the whole chloroplast genomes.

In the current work, the whole plastid DNA sequences of twenty-four *Pinus* species were used for the analysis of phylogeny. The reconstructed phylogenetic trees based on the maximum likelihood method, maximum parsimony, and Bayesian interference. The two major clades were recognized which included the subgenus *Strobus* (single needle section) and subgenus *Pinus* (double-needle section) of pine species (Fig. 4). The phylogenetic tree showed most of the monophyletic clade with high bootstrap value. The *P. pumila is* closely related to *P. sibirica* and *P. fenzeliana*.

### 3.6 Molecular dating

**Fig. 5:**
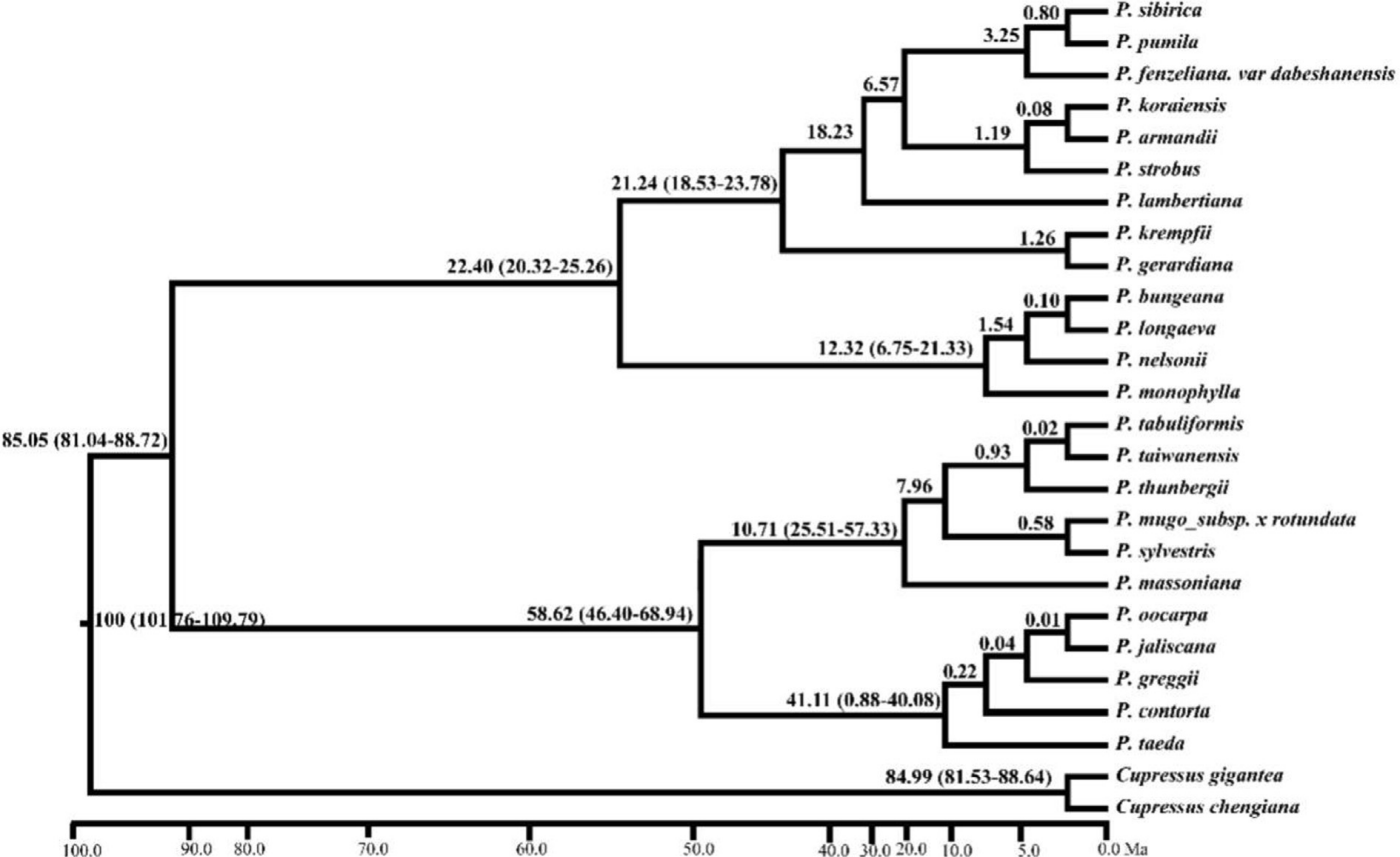
Chronogram for the *Pinus* species obtained using BEAST based on the cp genome.

The Beast molecular clock evaluated the divergence times in the genus *Pinus*. Molecular dating of the genus *Pinus* has instigated about 100 MYA (95% HPD, 101.76.35–109.79 MYA). The first divergence between the two subgenera (*Strobus* and *Pinus*) has originated at 85.05 MYA (95% HPD, 81.04–88.02 MYA). Subgenus *Strobus* diverged about 22.40 MYA (95% HPD, 20.32–25.26 MYA), and subgenus *Pinus* diverged about 58.62 MYA (95% HPD, 46.40–68.94 MYA) (Fig. 5).

## 4 Discussion

Taxonomic studies have used the plastid DNA to assess the closely related species of the *Pinus* species. The whole plastome of twenty-four genus *Pinus* were used to assess their phylogenetic relationship in the family Pinaceae. Land plants have an extremely well-maintained plastome, and four regions with altered cp genome sizes and length (Hansen et al., 2007, Plunkett and Downie, 2000, Qian et al., 2013). Besides, the overall GC contents of the (LSC and SSC) regions in all the *Pinus* species were higher than the IRs region. In addition, the *Pinus* plastid genome, the subgenus *Strobus* has the high GC content of *P. koraiensis* (38.8%), and subgenus *Pinus; P. massoniana* (38.6%). Subsequently, in the overall genus *Pinus* highest LSC was obtained for *P. bungeana* (38.1%), SSC *P. krempfii* (39.9 %) and IRs *P. gerardiana* (39.3%) regions. The relatively highly GC contents of the IRs region were regularly featured to the rRNA and tRNA genes (He et al., 2016, Shen et al., 2017). Generally, the large IRs play essential role in sustaining the constancy of the plastid genome (Wu et al., 2011). However, the loss of an extensive IRs result in few differences in the genome structures and gene content in the plastid genome (Yi et al., 20132013). There is no large IRs region in the complete plastome of the conifer’s species. In this study, we observed the IR regions in the subgenera (*Strobus* and *Pinus*) (326 to 487 bp). Generally, some differences in sequence size were also originated in the small IRs region among *Pinus* genome.

Previous studies suggested that the repetitive sequence variations played a significant role in the reorganization and maintenance of the cp genomes (Cavalier-Smith, 2002). Recently, we found that dispersed, palindromic, and tandem repeats in twenty-four *Pinus* species, demonstrated that dispersed repeats number is more palindromic whereas in tandem repeats was lower. Some repeat motifs were circulated in the intergenic spacer and intron regions, which were similar in preceding studies (Yang et al., 2016). The long repeat sequence might sustain the constancy of plastome, which were comparable to previous studies (Maréchal and Brisson, 2010). We identified a total of 769 SSRs from twenty-four *Pinus* species. The mononucleotide repeats were more frequent in the plastid genome, and they represented in 4.91% of the aggregate SSRs. Furthermore, the SSRs contain (1–6) nucleotide repeat motifs, which are generally dispersed in the whole genome and have an undue influence on the genome rearrangement and recombination (Ni et al., 2016). SSRs also has been identified in the highest number of *P. sibirica* and *P. fenzeliana* (47, 47). The highest SSRs was obtained for mono-, and di-nucleotide repeats, whereas in tri-, tetra-, penta, and *hexa*-nucleotide repeat sequences were lower in all *Pinus* species (Yu et al., 2017, Song et al., 2017). The SSRs result showed agreement with the previous work in which the mono-nucleotides were A/T, and all of the di-nucleotides were AT /TA repeats units and composed with the A/T-richness in the plastid genome (Han et al., 2015).

The *Pinus* plastome sequence was analyzed by the mVISTA program, as a reference with *P. bungeana* (Fig. S3). The comparative study of our results showed that the IRs region is less diverged than the (LSC and SSC) regions. Also, the non-coding regions are highly fluctuating than coding regions, displaying significant different regions among the *Pinus* plastome (Ni et al., 2016). Though, the divergent hotspot region includes eleven genes (*trnG-GCC, trnL-UAG, trnL-CAA, trnQ-UUG, rpoC1, rpoC2, psaC, ycf1, ycf2, chIL,* and *chlN*) in the non-coding regions. Moreover, among all twenty-four plastid genome sequences, the cp genome variations of higher plants were more conserved, and the plastid genome of *Pinus* species showed very low genetic divergence. The current results showed resemblance with previous studies (Qian et al., 2013), and revealed different coding regions in the *Pinus* species. Generally, the synonymous and non-synonymous nucleotide sites are beneficial for evolutionary studies and population genetics (OGAWA et al., 1999). In this study, we determined seven cp protein-coding genes that exposed site-specific selection (*matK, petD, psaI, rps18, psaM, ycf1,* and *ycf2*) for the *Pinus* species (Table S2). In the selective pressure analysis, we isolated a total of four types of photosynthesis gene groups, which are: 1. four genes’ subunits of photosystem I (*psaI, psaM, ycf1* and *ycf2*), 2. One small subunit of the ribosomal gene (*rps18*), 3. Subunit of cytochrome *b*/f complex (*petD*), and 4. One gene of maturaes (*matK*). In addition, a total of 11 genes observed with the encoded small subunit of the ribosome, in which only one gene of rps18 was found in the restricted positive selection. However, positively selected genes performed a significant function in the variation of the *Pinus* species under diverse environmental condition.

The complete chloroplast genome has been commonly used in the phylogeny of gymnosperm plants (Parks et al., 2012, Zhu et al., 2016). Based on evaluations of protein-coding genes (PCGs) some studies have discovered the phylogenetic analysis at the profound nodes (Moore et al., 2010, Eckert and Hall, 2006). These analyses enhanced our knowledge about the phylogenetic relationship and evolutionary studies among *Pinus* species. The current study is based on the phylogenetic investigation of the whole plastome sequence of twenty-four *Pinus* species, using *C. chengiana* and *C. gigantean* as outgroups. However, we obtained a phylogenetic tree with (ML, MP, and BI) methods (Fig. 3). Phylogenetic tree of genus *Pinus* was mainly separated into two different classes similar to single vascular needle and double vascular needle section plants. Among single needle section plants species, the *P. pumila* showed closed positioned with *P. fenzeliana,* and *P. sibirica* in the same clade, which has a close relationship with each other (Fig. 3). This finding determined the closest relationship among these species. In addition, our study has been recognized that *P. bungeana* and *P. gerardiana* have a close association with each other. Similar to this study, a previous study also demonstrated a closed position of *P. bungeana* and *P. gerardiana* species (Liu et al., 2014).

To evaluate the divergence time of genus *Pinus* the beast molecular clock evaluated the divergence times for *Pinus* species. The *Pinus* species have been instigated about 100 MYA (95% HPD, 101.76.35–109.79 MYA). The first divergence between the two subgenera of *Strobus* and subgenera *Pinus* occurred about 85.05 MYA (95% HPD, 81.04–88.02 MYA). Subgenus *Strobus* diverged about 22.40 Mya (95% HPD, 20.32–25.26 Mya), and subgenus *Pinus* diverged about 58.62 Mya (95% HPD, 46.40–68.94 MYA) (Fig. 4). These results were also broadly dependable with the previously fossil histories from the early Cretaceous. Similar to our study, the molecular dating of the previous study also obtained comparable results (Liu et al., 2014).

## 5 Conclusion

In present investigation, the evidence of the whole chloroplast genome of *Pinus* species. We compared their whole plastid genomes developed by plentiful genetic resources, comprised hotspots region and SSRs. Plastid DNA had a distinctive circular form with a preserved genome prearrangement. The molecular study of plastome in the genus *Pinus* also provided the phylogenetic relationship and molecular dating. The cp genome structure and genetic resources showed that the study will enhance our understanding of phylogeny, conservation and population genetics.

## Funding

The publication of the present work is supported by the Natural Science Basic Research Program of Shaanxi Province (grant no. 2018JQ5218) and the National Natural Science Foundation of China (51809224), Top Young Talents of Shaanxi Special Support Program.

## Declaration of Competing Interest

The authors declare that they have no known competing financial interests or personal relationships that could have appeared to influence the work reported in this paper.
