# Supplementary material for: Comparative genome sequence and phylogenetic analysis of chloroplast for evolutionary relationship among Pinus species
Source: Saudi J Biol Sci. 2021 Nov 12;29(3):1618–27. doi: 10.1016/j.sjbs.2021.10.070 (PMC8913380; doi:10.1016/j.sjbs.2021.10.070)
Supplement: Supplementary data 1 [file mmc1.docx]

**Supplementary Materials**.

**Table S1**: Base compositions of chloroplast genomes of twenty-four *Pinus* species.

| **Section** | **Species** | **LSC%** | **SSC%** | **IR%** |
| --- | --- | --- | --- | --- |
| Subgenus strobus (Single needle section) |  |  |  |  |
|  | *P. armandii* | 38.0 | 39.8 | 37.0 |
|  | *P. bungeana* | 38.1 | 39.8 | 37.9 |
|  | *P. fenzeliana* | 38.0 | 39.9 | 36.8 |
|  | *P. gerardiana* | 38.1 | 39.6 | 39.3 |
|  | *P. koraiensis* | 38.0 | 39.8 | 38.1 |
|  | *P. krempfii* | 38.0 | 39.9 | 36.2 |
|  | *P. lambertiana* | 38.0 | 39.7 | 36.9 |
|  | *P. monophylla* | 37.9 | 39.6 | 38.1 |
|  | *P. nelsonii* | 37.9 | 39.7 | 36.0 |
|  | *P. pumila* | 38.0 | 39.7 | 36.5 |
|  | *P. sibirica* | 38.0 | 39.7 | 39.6 |
|  | *P. strobus* | 38.0 | 39.7 | 37.3 |
|  | *P. longaeva* | - | - | - |
| Subgenus *Pinus* (Double needle section |  |  |  |  |
|  | *P. massoniana* | 37.9 | 39.4 | 35.5 |
|  | *P. mugo* | 37.9 | 39.3 | 36.3 |
|  | *P. sylvestris* | 37.9 | 39.5 | 39.3 |
|  | *P. tabuliformis* | 37.9 | 39.4 | 35.6 |
|  | *P. taeda* | 38.0 | 39.2 | 36.0 |
|  | *P. taiwanensis* | 37.9 | 39.4 | 37.0 |
|  | *P. thunbergii* | 37.9 | 39.4 | 35.6 |
|  | *P.oocarpa* | 37.9 | 39.2 | 35.5 |
|  | *P. greggii* | 37.9 | 39.2 | 36.0 |
|  | *P. jaliscana* | 37.9 | 39.2 | 36.0 |
|  | *P. contorta* | 37.9 | 39.2 | 36.8 |

**Table S2:** Likelihood ratio test (LRT) of the variable w ratio under different models.

| **Gene** | **comparisons** | **2Δl** | **df** | **p** |
| --- | --- | --- | --- | --- |
| *matK* | M0 vs M3 | 306.24839 | 4 | 4.356654e-32 |
|  | M1 vs M2 | 104.616551 | 2 | 4.379321e-12 |
|  | M7 vs M8 | 25.174838 | 2 | 0.001847892 |
| *petD* | M0 vs M3 | 33.206282 | 4 | 0.002307969 |
|  | M1 vs M2 | 22.560708 | 2 | 0.003552245 |
|  | M7 vs M8 | 22.864272 | 2 | 0.003292632 |
| *psaI* | M0 vs M3 | 92.26554 | 4 | 2.311038e-09 |
|  | M1 vs M2 | 59.04017 | 2 | 3.888615e-07 |
|  | M7 vs M8 | 50.18537 | 2 | 3.557891e-06 |
| *rps18* | M0 vs M3 | 210.8675 | 4 | 6.846409e-22 |
|  | M1 vs M2 | 0 | 2 | 1 |
|  | M7 vs M8 | 54.497888 | 2 | 1 |
| *ycf1* | M0 vs M3 | 3356.53658 | 4 | 0 |
|  | M1 vs M2 | 360.1228 | 2 | 7.946279e-40 |
|  | M7 vs M8 | 363.4309 | 2 | 3.475293e-40 |
| *ycf2* | M0 vs M3 | 2856.30952 | 4 | 5.426093e-308 |
|  | M1 vs M2 | 561.6866 | 2 | 1.036698e-61 |
|  | M7 vs M8 | 355.67584 | 2 | 2.415385e-39 |
| *psaM* | M0 vs M3 | 24.176832 | 4 | 0.01670575 |
|  | M1 vs M2 | 16.985278 | 2 | 0.01431683 |
|  | M7 vs M8 | 18.989302 | 2 | 0.008674865 |


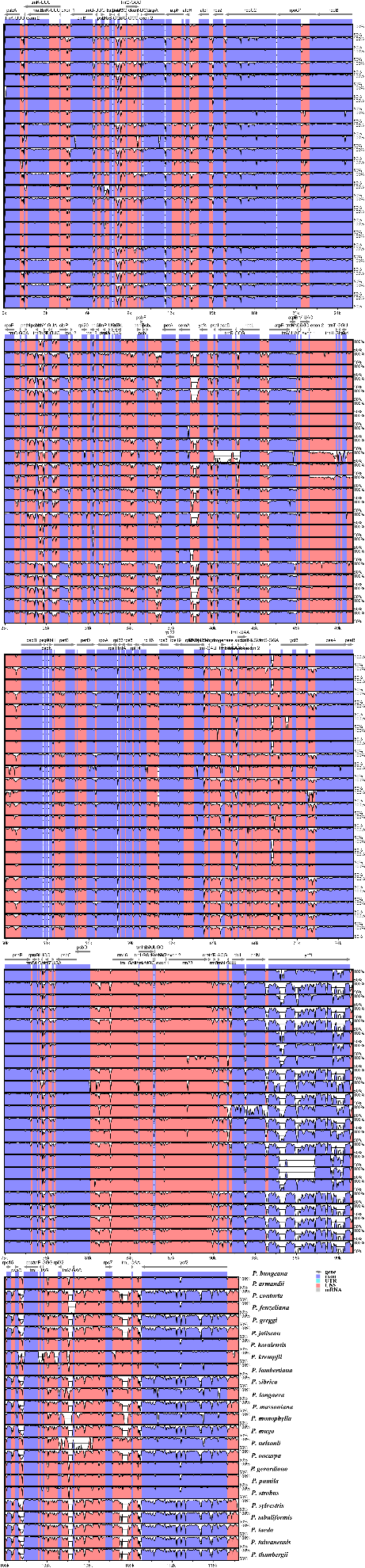


**Fig S1**

**Figure S1**: Genome regions are colour-coded as protein-coding, rRNA coding, tRNA coding or conserved non-coding sequences.
